# Development and validation of a risk prediction model for demoralization syndrome in patients with chronic heart failure

**DOI:** 10.3389/fmed.2026.1904460

**Published:** 2026-09-30

**Authors:** Ting Yao, Qiao Hu, Xianqing Yang, Yanyan Shao, Shinan Yu, Zheyuan Xia, Xiaoqing Yang, Xiang Wang

**Affiliations:** 1 School of Nursing, Anhui University of Chinese Medicine, Hefei, Anhui, China; 2 Key Laboratory of Geriatric Nursing and Heath, Anhui University of Chinese Medicine, Hefei, China; 3 Department of Plastic and Reconstructive Surgery, The Second Affiliated Hospital of Anhui Medical University, Hefei, China

**Keywords:** chronic heart failure, demoralization syndrome, influencing factors, predictive model, traditional Chinese medicine constitution

## Abstract

**Objective:**

To investigate the associated factors for demoralization syndrome in patients with chronic heart failure (CHF) and to develop a risk prediction model that facilitates early identification of high-risk individuals.

**Methods:**

A total of 331 patients with CHF were recruited from two hospitals in Anhui Province, China, between March 2025 and February 2026 using convenience sampling. Demographic and clinical data were collected, and validated scales were used to assess demoralization, social support, and symptom burden. Univariate and multivariable logistic regression analyses were performed—specifically, univariate analysis was employed to screen for associated factors, while multivariable regression was applied to identify independent predictors and construct the prediction model. Model performance was assessed using the area under the receiver operating characteristic curve (AUC), calibration curves, and decision curve analysis.

**Results:**

Demoralization syndrome was identified in 57.7% of patients. In the univariate analyses, several variables emerged as associated factors. In the multivariable model, exercise frequency, NYHA functional class, SSRS score, KCCQ score, and blood stasis constitution were identified as independent predictors and were incorporated into the final prediction model. The AUC was 0.835 (95% CI: 0.783–0.886) in the training set and 0.822 (95% CI: 0.735–0.909), in the validation set. The model showed good calibration and favorable clinical net benefit.

**Conclusion:**

Exercise frequency, NYHA functional class, social support, symptom burden, and blood stasis constitution are independent predictors of demoralization syndrome in patients with CHF. The proposed prediction model demonstrated robust discrimination and calibration in internal validation, suggesting its potential as a preliminary auxiliary reference for risk awareness.

## 1 Introduction

Chronic heart failure represents the terminal stage of various cardiovascular diseases and is characterised by a high incidence, high mortality and high readmission rates. In addition to progressive cardiac dysfunction, patients face multiple pressures, including symptom-related distress, uncertainty about treatment, and the financial burden of long-term medication use (1). Furthermore, patients with chronic heart failure often present with a complex state of multimorbidity and holistic frailty (2) A qualitative interview study involving patients with chronic heart failure found that these factors can trigger various negative emotional responses (3). Without timely and effective intervention, patients may struggle to regulate their emotions, leading to poorer treatment adherence and reduced self-management capacity, which may ultimately adversely affect disease outcomes (4).

Among various negative psychological states, demoralization syndrome has gradually attracted attention in recent years. This syndrome is characterised by a subjective sense of helplessness, hopelessness, failure, and a lack of coping skills; it is prevalent across a wide range of chronic conditions and has an adverse impact on clinical outcomes (5, 6). Patients exhibiting this syndrome often demonstrate reduced treatment adherence, diminished self-management skills and a weakened willingness to actively participate in rehabilitation. This leads to poor disease control, an increased risk of readmission and a significant decline in quality of life. It is worth noting that a vicious cycle may exist between demoralization syndrome and disease progression: the symptom burden and functional limitations caused by the disease exacerbate the patient's sense of hopelessness, while the persistent state of demoralization syndrome further weakens the patient's ability to cope with the disease, ultimately affecting overall survival outcomes.

Currently, predictive research on demoralization syndrome in patients with chronic heart failure remains limited. A small number of international studies have explored the relationship between demoralization syndrome and heart failure prognosis, but these have largely focused on descriptive analyses or cross-sectional association studies, with a lack of systematic attempts to construct risk prediction models. Domestically, this field is still in its infancy, with a particular lack of simple, practical predictive tools suitable for the early clinical identification of high-risk individuals. Although existing psychological assessment scales can be used to evaluate negative emotions, they are often time-consuming, rely on professionals for interpretation, and face limitations in their implementation within primary care settings. Therefore, exploring more integrated and actionable predictive factors and developing easily applicable predictive models holds significant clinical importance.

Traditional Chinese Medicine (TCM) constitutional theory provides a potential approach for early identification and intervention of demoralization syndrome. Constitution refers to an individual's relatively stable characteristics in morphology, physiology, and psychology, shaped by both innate endowment and acquired factors (7). Different constitutional types exhibit varying susceptibilities to specific diseases (8, 9), and constitutional imbalances are significant risk factors for various conditions. Notably, constitution is dynamically adjustable, offering a theoretical basis for “identification and regulation” of constitutional types.

For patients with chronic heart failure, impaired cardiac function and limitations in daily activities, coupled with progressively worsening symptoms as the disease progresses, exert a continuous impact on their physiological, psychological and social roles, making them more prone to negative emotions. Therefore, constructing a predictive model based on Traditional Chinese Medicine constitution theory will help identify key factors influencing the development of demoralization syndrome in patients with chronic heart failure, thereby providing a scientific basis for formulating targeted treatment and intervention measures.

In summary, this study intends to employ convenience sampling to recruit eligible patients with chronic heart failure from two hospitals in Anhui Province. General and clinical data will be collected, and various scales will be used to assess demoralization syndrome, social support, psychological resilience, anxiety and depression, and symptom burden. Through univariate and multivariate logistic regression analyses, a risk prediction model for demoralization syndrome will be constructed, and the model's discriminatory power, calibration, and clinical net benefit will be evaluated. The aim is to provide a theoretical basis and practical tools for the early clinical identification of high-risk individuals, the implementation of precise prevention, and timely intervention.

## 2 Methods

### 2.1 Study design and population

This study employed a cross-sectional survey design. Using convenience sampling, 331 patients with chronic heart failure (CHF) who met the inclusion and exclusion criteria and were hospitalized in the cardiovascular departments of two hospitals in Anhui Province between March 2025 and February 2026 were recruited as study subjects. Data were collected via a questionnaire. Informed consent was obtained from all participants prior to the start of the study. This study has been approved by the Ethics Committee. (Ethics Approval Number: AHUCM-HSS-2024012).

Inclusion Criteria: (1) Meet the diagnostic criteria outlined in the “Chinese Guidelines for the Diagnosis and Treatment of Heart Failure 2024” (10): ① History of coronary atherosclerotic heart disease, pulmonary rales, hypertension, or edema of both lower limbs; ② Presence of high-risk factors for heart failure: cardiac murmur, use of diuretics, jugular vein distension, orthopnea/paroxysmal nocturnal dyspnea, lateral displacement of the apical impulse, or difficulty in diffusion; ③ Pulmonary congestion and edema on chest x-ray, and ECG abnormalities indicating cardiac enlargement; ④ Natriuretic peptide levels: NT-proBNP ≥ 125 ng/L or BNP > 35 ng/L; ⑤ Abnormal cardiac structure and/or function on echocardiography. (2) Age ≥ 18 years; (3) Ability to communicate effectively; (4) The subject has full cognitive and behavioral capacity;

Exclusion criteria: (1) Concurrent malignant tumors (any site) or severe complications such as decompensated failure of vital organs (e.g., liver or kidney); (2) Mental illness or inability to communicate.

### 2.2 Sample size estimation

According to Kendall's method for estimating sample size in multiple regression (11), the minimum sample size should be 5 to 10 times the number of independent variables, This study utilized 7 survey instruments, comprising a total of 50 variables (34 variables from the General Information Questionnaire, 2 variables from the Chinese Version of the Demoralization Scale II, 3 variables from the Social Support Rating Scale, and 2 variables from the Kansas City Cardiomyopathy Questionnaire, and 9 variables from the Traditional Chinese Medicine Constitution Scale). To account for invalid assessments due to incomplete data and other factors, a 10% margin was added to the sample size to mitigate the risk of attrition. The sample size formula was [Max(number of dimensions) × (5–10)] × (1 + 10%). The minimum required sample size was 275 cases. The final valid sample size included in this study was 331 cases.

### 2.3 Variables and instruments

Based on a literature review, relevant domestic and international research reports, and actual clinical conditions, we summarized sociodemographic factors that may influence the occurrence of CHF and designed a questionnaire for general demographic data. The questionnaire included the following items: (1) General demographic data: age, gender, marital status, educational level, place of residence, housing status, occupation, monthly per capita household income, health insurance coverage, smoking history, and alcohol consumption history. (2) Medical record data: hemoglobin, T3, T4, TSH, BNP, NT-proBNP, C-reactive protein, serum creatinine, blood glucose, serum potassium, serum sodium, blood urea nitrogen, cardiac troponin, duration of heart failure, presence of complications, treatment methods, number of hospitalizations in the past year, and history of medication use.

#### 2.3.1 Depression scale II

The DS-II was developed in 2016 by Australian scholars Robinson et al. (12). as a refinement of the Demoralization Scale (DS). The Chinese version was translated by Ou Na et al. (13). The DS-II consists of two dimensions—Meaning and Purpose, and Distress and Coping—and 16 items, and is used to assess an individual's level of demoralization. The total score ranges from 0 to 32 points, with scores ≤9 indicating mild demoralization, 10∼19 indicating moderate demoralization, and >19 indicating severe demoralization.Consistent with previous studies (14, 15), a score >9 was defined as moderate-to-severe demoralization and served as the positive screening threshold in this study. In this study, the Cronbach's *α* coefficient for this scale was 0.93.

#### 2.3.2 Social support scale

The Social Support Scale, developed and revised by Xiao Shuiyuan (16), consists of 10 items across three dimensions: subjective support, objective support, and utilization of support. Items 1–4 and 8–10 are scored from 1 to 4 points, ranging from “no support” to “full support.” Item 5 consists of four sub-items (a, b, c, and d), each scored from 1 to 4 points, ranging from “none” to “full support.” The total score is the sum of these scores. For items 6 and 7, a score of 0 is assigned if “no sources” are reported; otherwise, points are awarded based on the number of sources. The total score of the scale is the sum of the item scores; a higher score indicates a higher level of social support received by the patient. A total score of ≤22 is classified as low, 23–44 as moderate, and 45–66 as high. In this study, the Cronbach's *α* coefficient for this scale was 0.71.

#### 2.3.3 Kansas City cardiomyopathy questionnaire

The Kansas City Cardiomyopathy Questionnaire (KCCQ) was first published in 2000 by a team led by Dr. John Spertus at the University of Missouri-Kansas City and is widely used in clinical research to assess heart failure (17). It is primarily based on patients’ experiences and their perceptions of how heart failure affects various aspects of their lives. The KCCQ uses a two-week recall period and covers 23 questions across seven domains: symptom frequency, symptom burden, symptom stability, physical limitations, social limitations, quality of life, and self-efficacy. These are organized into two dimensions: overall well-being and symptoms. The questionnaire uses a 0–100 scoring system, where lower scores indicate more severe symptoms and greater limitations. Scores of 0–24 indicate very poor to poor health, 25–49 indicate poor to fair health, 50–74 indicate fair to good health, and scores of 75 or higher represent good to excellent health. In this study, the Cronbach's *α* coefficient for this scale was 0.91.

#### 2.3.4 Constitutional types in traditional Chinese medicine

According to the standards set forth in the “Classification and Determination of Constitutional Types in Traditional Chinese Medicine” (ZYYXH/T 157–2009) published by the Chinese Association of Traditional Chinese Medicine in 2009 (18), participants complete the “Traditional Chinese Medicine Constitutional Classification and Determination Form.” Each question is scored on a 5-point scale to calculate the raw score and the converted score, and the constitutional type is determined based on the standards: Raw score = the sum of the scores for each item; Converted score = (Raw score—Number of items)/(Number of items × 4) × 100. Based on the Traditional Chinese Medicine Constitution Classification Table, constitutions are classified as Harmonious Constitution, Qi Deficiency Constitution, Yang Deficiency Constitution, Yin Deficiency Constitution, Phlegm-Dampness Constitution, Damp-Heat Constitution, Blood Stasis Constitution, Qi Stagnation Constitution, and Special Constitution. Except for the Harmonious Constitution, all others are considered Imbalanced Constitutions. For patients with mixed constitutions (i.e., those with conversion scores of ≥40 for two or more imbalanced constitutions), this study designates the constitution with the highest conversion score as the patient's primary constitution type to investigate the association between the primary constitution and demoralization syndrome.

### 2.4 Data collection

This study employed a structured questionnaire survey. Prior to the formal survey, the relevant questionnaires were prepared, and all research staff received standardized training to ensure consistent use of instructions during the survey. During the formal survey, the interviewer first explained the purpose and significance of the survey to the patient. After obtaining the patient's consent, the questionnaire was distributed. For patients with low educational levels or those unable to answer on their own, the interviewer read the questions aloud and assisted in completing the form based on the patient's responses. After the questionnaire was completed, it was checked for any omissions or errors, which were corrected promptly, and the questionnaire was collected on the spot. Data was entered into Excel by two independent interviewers. After data entry was completed, the entries were cross-checked; any discrepancies were promptly verified against the original questionnaire and corrected to ensure data accuracy.

To minimize potential bias in model construction, data collection and entry were conducted by one group of investigators who were blinded to the research hypotheses. Statistical analyses were performed independently by a separate group of investigators who only accessed de-identified data and were blinded to participant identities and all personally identifiable information throughout the analysis.

### 2.5 Statistical analysis

Statistical analysis of the data was performed using SPSS 26.0. Quantitative data were tested for normality using the Kolmogorov–Smirnov test; data that followed a normal distribution were expressed as mean ± standard deviation ($\bar{x}$± S). Independent samples t-tests were used for comparisons between groups, while data that did not follow a normal distribution were expressed as median and quartiles (M (*P*_25_, *P*_75_), and the Mann–Whitney U test was used for comparisons between groups. Categorical variables were described using frequency and proportion [n (%)], and chi-square tests were used for between-group comparisons. The presence or absence of demoralization syndrome was used as a binary dependent variable. Variables were screened using univariate analysis, and independent variables with statistical significance in the univariate analysis (*P* < 0.05) were included in a multivariate logistic regression analysis. Prior to model construction, the Box-Tidwell test was performed to assess the linearity assumption between all continuous independent variables and the logit. The backward selection method was used to identify the final variables included in the model and to construct the model. To evaluate model performance and correct for overfitting bias, internal validation was performed using bootstrap resampling with optimism correction, implemented via the validate() function in the rms R package. The optimism-corrected C-index, calibration slope, calibration intercept, and Brier score were calculated to assess discrimination and calibration. Using R version 4.4.1, the dataset was split into a 7:3 ratio using random stratification for internal validation, and the model's stability was assessed using the Bootstrap method. The model's discriminatory ability was assessed by plotting ROC curves and calculating the area under the curve (AUC). A calibration curve was used to visually demonstrate the consistency between the model's predicted probabilities and actual event probabilities, thereby evaluating its calibration. Decision curve analysis (DCA) was employed to evaluate the net benefit of the model at different threshold probabilities, thereby assessing its clinical applicability. This study followed the TRIPOD statement for reporting prediction model studies.

## 3 Results

### 3.1 Characteristics of the participants

Based on the inclusion and exclusion criteria, a total of 345 questionnaires were distributed in this study. After excluding invalid questionnaires with incomplete entries or answers showing obvious patterns, 331 questionnaires were recovered, resulting in a valid response rate of 96.23%. “Presence of Demoralization Syndrome” was treated as a binary variable: subjects with a DS-II score ≤ 9 were classified into the non-Demoralization Syndrome group, while those with a DS-II score > 9 were classified into the Demoralization Syndrome group. Among them, 191 cases (57.7%) were in the CHF combined with Demoralization Syndrome group, and 140 cases (42.3%) were in the CHF group.

### 3.2 Results of the univariate analysis

Univariate analysis revealed that age, income level, smoking history, alcohol consumption history, daily physical activity, primary caregiver, educational level, number of hospitalizations in the past year, BMI classification, cardiac function classification, BNP, NT-proBNP, SSRS score, KCCQ score, and presence of blood stasis constitution were significant factors (all *P* < 0.05). See Table 1 for details.

**Table 1:** Univariate analysis of demoralization syndrome in CHF patients.

Variable	Without demoralization (*n* = 140)	With demoralization (*n* = 191)	*Z*/*χ*2 value	*P*-value
Age, n(%)			V = 0.164	0.010
<45 years	2 (1.429)	4 (2.094)		
45∼60 years	23 (16.429)	12 (6.283)		
>60 years	115 (82.143)	175 (91.623)		
Gender, n(%)			χ²=2.753	0.097
Male	73 (52.143)	82 (42.932)		
Female	67 (47.857)	109 (57.068)		
Marital status, n(%)			V = 0.113	0.275
Married	118 (84.286)	147 (76.963)		
Widowed	20 (14.286)	38 (19.895)		
Divorced	0 (0.00)	3 (1.571)		
Unmarried	2 (1.429)	3 (1.571)		
Occupation, n(%)			χ²=7.126	0.129
Retired	72 (51.429)	108 (56.545)		
Unemployed	39 (27.857)	57 (29.843)		
Farmer	15 (10.714)	19 (9.948)		
Self-employed	10 (7.143)	3 (1.571)		
Company employee	4 (2.857)	4 (2.094)		
Residence, n(%)			χ²=0.364	0.834
Urban	96 (68.571)	134 (70.157)		
Township	12 (8.571)	13 (6.806)		
Rural	32 (22.857)	44 (23.037)		
Medical insurance, n(%)			V = 0.115	0.264
Employee basic medical insurance	74 (52.857)	110 (57.592)		
Resident medical insurance	62 (44.286)	76 (39.791)		
New Rural Cooperative	4 (2.857)	2 (1.047)		
Other	0 (0.00)	3 (1.571)		
Monthly income (RMB), n(%)			χ²=7.927	0.019
>5000	38 (27.143)	77 (40.314)		
2000∼5000	92 (65.714)	96 (50.262)		
<2000	10 (7.143)	18 (9.424)		
Smoking, n(%)			χ²=5.348	0.021
Yes	31 (22.143)	24 (12.565)		
No	109 (77.857)	167 (87.435)		
Alcohol consumption, n(%)			χ²=7.578	0.006
Yes	26 (18.571)	16 (8.377)		
No	114 (81.429)	175 (91.623)		
Regular exercise, n(%)			χ²=33.155	<0.001
Daily	45 (32.143)	23 (12.042)		
Occasionally	33 (23.571)	25 (13.089)		
Almost never	62 (44.286)	143 (74.869)		
Primary caregiver, n(%)			V = 0.198	0.009
Spouse	80 (57.143)	79 (41.361)		
Children	57 (40.714)	94 (49.215)		
Professional caregiver	2 (1.429)	6 (3.141)		
None	1 (0.714)	8 (4.188)		
Other family	0 (0.00)	4 (2.094)		
Education level, n(%)			χ²=9.921	0.042
Illiterate	30 (21.429)	39 (20.419)		
Primary school	38 (27.143)	53 (27.749)		
Junior high school	42 (30.000)	42 (21.990)		
High school	22 (15.714)	26 (13.613)		
College & above	8 (5.714)	31 (16.230)		
Heart failure duration, n(%)			χ²=1.380	0.502
<1 year	42 (30.000)	49 (25.654)		
1∼3 years	22 (15.714)	26 (13.613)		
>3 years	76 (54.286)	116 (60.733)		
Hospitalizations (past year), n(%)			χ²=4.148	0.042
1∼2 times	114 (81.429)	137 (71.728)		
≥3 times	26 (18.571)	54 (28.272)		
NYHA functional class, n(%)			χ²=15.457	<0.001
Class I & II	54 (38.571)	44 (23.037)		
Class III	72 (51.429)	101 (52.880)		
Class IV	14 (10.000)	46 (24.084)		
Acute cardiovascular event (last 2 months), n(%)			χ²=0.040	0.841
Yes	18 (12.857)	26 (13.613)		
No	122 (87.143)	165 (86.387)		
Number of comorbidities, n(%)			χ²=1.025	0.311
1∼2	59 (42.143)	70 (36.649)		
≥3	81 (57.857)	121 (63.351)		
Daily medications, n(%)			χ²=0.008	0.930
1∼2 types	93 (66.429)	126 (65.969)		
≥3 types	47 (33.571)	65 (34.031)		
Treatment regimen, n(%)			χ²=5.744	0.057
Medication only	111 (79.286)	134 (70.157)		
Medication + coronary stent	26 (18.571)	43 (22.513)		
Medication + pacemaker	3 (2.143)	14 (7.330)		
BMI (kg/m²), n(%)			χ²=7.548	0.023
<18.5	17 (12.143)	46 (24.084)		
18.6∼23.9	100 (71.429)	116 (60.733)		
≥24	23 (16.429)	29 (15.183)		
Hemoglobin (g/L) M (P_25_,P_75_)	127.00 (111.50, 136.50)	123.50 (110.75, 131.75)	Z = −0.86	0.388
T3 (nmol/L)M(P_25_,P_75_)	3.84 (3.32, 4.24)	3.74 (3.41, 4.05)	Z = −0.46	0.643
T4 (nmol/L)M(P_25_,P_75_)	13.03 (12.03, 14.03)	13.30 (12.15, 14.09)	Z = −0.22	0.823
TSH (mIU/L)M(P_25_,P_75_)	2.36 (1.28, 3.30)	2.64 (1.57, 3.57)	Z = −0.96	0.335
BNP (pg/mL)M(P_25_,P_75_)	143.00 (66.60, 505.80)	675.50 (208.35, 1435.50)	Z = −2.35	0.019
NT-proBNP (pg/mL) M (P_25_,P_75_)	2653.00 (809.00, 4587.00)	272.62 (112.62, 1340.03)	Z = −3.00	0.003
C-reactive protein (mg/L)M (P_25_,P_75_)	3.08 (2.10, 13.97)	3.57 (1.67, 4.84)	Z = −0.47	0.635
Serum creatinine (µmol/L)M (P_25_,P_75_)	71.80 (60.35, 93.10)	76.80 (63.80, 99.50)	Z = −0.70	0.481
Blood glucose (mmol/L)M (P_25_,P_75_)	4.99 (4.71, 6.04)	5.29 (4.60, 6.27)	Z = −0.10	0.921
Serum potassium (mmol/L)M (P_25_,P_75_)	4.10 (3.86, 4.26)	4.13 (3.68, 4.38)	Z = −0.13	0.893
Serum sodium (mmol/L)M (P_25_,P_75_)	140.90 (139.40, 143.20)	141.05 (138.94, 142.88)	Z = −0.04	0.965
Blood urea nitrogen (mmol/L)M (P_25_,P_75_)	6.30 (5.28, 8.84)	6.91 (5.07, 8.35)	Z = −0.32	0.747
Cardiac troponin (ng/mL)M (P_25_,P_75_)	0.03 (0.01, 0.03)	0.03 (0.01, 0.03)	Z = −1.10	0.273
SSRS scoreM (P_25_,P_75_)	34.00 (31.00, 36.00)	30.00 (28.00, 32.00)	Z = −7.38	<0.001
KCCQ scoreM (P_25_,P_75_)	56.00 (49.00, 64.00)	53.00 (47.00, 59.00)	Z = −2.45	0.014

### 3.3 Univariate analysis of constitutional types in traditional Chinese medicine

With the grouping variable as the dependent variable, the “peaceful” constitution—classified as a normal constitution type—was excluded from the scope of this study's analysis. The remaining eight constitutions were treated as independent variables, with a uniform coding scheme of “yes = 1, no = 0” for each factor. The results of univariate logistic regression analysis indicate that the difference in blood stasis constitution between the group with demoralization syndrome and the group without demoralization syndrome is statistically significant (*P* < 0.05). See Table 2 for details.

**Table 2:** Univariate analysis of TCM constitutions in CHF patients.

TCM Constitution	*β*	S.E	*Z*	*P*	OR (95%CI)
Qi deficiency	−0.07	0.26	−0.26	0.797	0.94 (0.57∼1.55)
Yang deficiency	−0.24	0.30	−0.80	0.426	0.79 (0.44∼1.42)
Yin deficiency	−0.59	0.45	−1.30	0.194	0.55 (0.23∼1.35)
Phlegm-dampness	−0.64	0.36	−1.80	0.072	0.53 (0.26∼1.06)
Dampness-heat	−0.60	0.48	−1.25	0.210	0.55 (0.22∼1.40)
Blood stasis	0.85	0.32	2.71	0.007	2.35 (1.27∼4.36)
Qi stagnation	1.90	1.06	1.80	0.072	6.67 (0.84∼52.75)
Special constitution	0.66	0.83	0.80	0.422	1.94 (0.38∼9.77)

### 3.4 Use logistic regression to select predictors

Using the presence of demoralization syndrome in patients with CHF as the dependent variable, and the 15 variables from the univariate analysis with *P* < 0.05 as independent variables—all with the lowest score serving as the reference group—we performed a logistic regression analysis using the backward LR method (a stepwise regression method that sequentially removes variables with the smallest contribution to the model). For details on the scoring method, see Table 3.

**Table 3:** Assignment of independent variables.

Variable	Assignment
Age	<45 = 1, 45–60 = 2, >60 = 3
Monthly income (RMB)	>5000 = 1, 2000–5000 = 2, <2000 = 3
Smoking history	Yes = 1, No = 2
Alcohol consumption history	Yes = 1, No = 2
Daily exercise status	Daily = 1, Occasionally = 2, Almost neve*r* = 3
Primary caregiver	Spouse = 1, Children = 2, Professional caregive*r* = 3, None = 4, Other family membe*r* = 5
Education level	Illiterate = 1, Primary school = 2, Junior high school = 3, High school = 4, College or above = 5
Number of hospitalizations in the past year	1–2 times = 1, ≥3 times = 2
BMI category	<18.5 = 1, 18.5–23.9 = 2, ≥24 = 3
NYHA functional class	I and II = 1, III = 2, IV = 3
BNP (pg/mL)	Raw value entered
NT-proBNP (pg/mL)	Raw value entered
SSRS score	Raw value entered
KCCQ score	Raw value entered
Blood stasis constitution (TCM)	Yes = 1, No = 2

### 3.5 Logistic regression analysis

Prior to multivariable logistic regression, the Box-Tidwell test was performed to assess the linearity assumption between all continuous independent variables and the logit. The results confirmed that all continuous variables satisfied the linearity assumption (SSRS: *P* = 0.310; KCCQ: *P* = 0.085; all *P* > 0.05). Multicollinearity was examined using the variance inflation factor (VIF), with the generalized VIF (GVIF) applied for polytomous variables. The adjusted GVIF^[1/(2×Df)] values for all predictors ranged from 1.038 to 1.863, all well below the recommended threshold of 2.0, indicating no substantial multicollinearity. Accordingly, all five variables were entered into the final multivariable logistic regression model. The results of multivariate logistic regression analysis showed that exercise frequency, NYHA class, SSRS scores, KCCQ scores, and blood stasis constitution were independent predictors for the development of demoralization syndrome in patients with CHF. Regarding exercise frequency, compared with those who exercised daily, the risk of developing demoralization syndrome was significantly higher among those who almost never exercised (OR = 3.47, 95% CI: 1.43–8.39, *P* = 0.006); however, the difference between those who exercised occasionally and those who exercised daily was not statistically significant (*P* > 0.05). Regarding NYHA class, compared with patients with NYHA class I/II, those with class IV had a significantly increased risk (OR = 7.91, 95% CI: 2.07–30.17, *P* = 0.002), while the difference between class III and class I/II was not statistically significant (*P* > 0.05). Regarding scale scores, higher SSRS scores (OR = 0.80, 95% CI: 0.74–0.87, *P* < 0.001) and higher KCCQ scores (OR = 0.96, 95% CI: 0.92–0.99, *P* = 0.042) were protective factors against the development of demoralization syndrome in patients with CHF. Regarding TCM constitution, compared with patients without the blood stasis constitution, those with the blood stasis constitution had a significantly increased risk of developing demoralization syndrome (OR = 2.88, 95% CI: 1.23–6.72, *P* = 0.014), suggesting that the blood stasis constitution is an independent predictor for demoralization syndrome in patients with CHF. See Table 4.

**Table 4:** Logistic regression analysis of demoralization syndrome.

Factor	β	S.E	Wald χ2	*P*	OR (95%CI)
Exercise (Reference: Daily)	—	—	—	—	1.00 (Reference)
Occasionally	0.20	0.55	0.14	0.713	1.22 (0.42∼3.58)
Almost never	1.24	0.45	7.62	0.006	3.47 (1.43∼8.39)
NYHA class (Reference: Ⅰand Ⅱ)	—	—	—	—	1.00 (Reference)
Ⅲ	0.07	0.39	0.03	0.853	1.08 (0.50∼2.33)
Ⅳ	2.07	0.68	9.18	0.002	7.91 (2.07∼30.17)
SSRS score	−0.22	0.04	24.9	<0.001	0.80 (0.74∼0.87)
KCCQ score	−0.04	0.02	4.12	0.042	0.96 (0.92∼0.99)
Blood stasis constitution (Reference: No)	—	—	—	—	1.00 (Reference)
Yes	1.06	0.43	6	0.014	2.88 (1.23∼6.72)

### 3.6 Construction and internal validation of risk prediction model

Based on the results of logistic regression analysis, a risk prediction model for the development of demoralization syndrome in patients with chronic heart failure (CHF) was constructed using exercise frequency, NYHA class, social support scores, Kansas City Cardiomyopathy Questionnaire scores, and the presence of blood stasis constitution as independent variables, and the occurrence of demoralization syndrome as the outcome variable. The model was developed based on the partial regression coefficients of the predictive factors. The specific predictive model formula is:

$$\begin{array}{rl} \mathrm{Logit}(P) & =8.42+1.24\times \mathrm{Exercise}\\ & +2.07\times \mathrm{NYHA}\;\mathrm{class}+1.06\times \mathrm{Blood}\;\mathrm{stasis}\;\mathrm{constitution}\text{-}0.22\\ \times SSRS\;score \end{array}\text{-}0.04\times \mathrm{KCCQ}\;\mathrm{score}$$

Using R 4.4.1 software, a nomogram was created to visualize the logistic regression model incorporating the above five independent predictors; the specific results are shown in Figure 1. Based on the *β* values of each predictor corresponding to the outcome variable in the model, corresponding scores are assigned to different value levels of each variable (the value levels of each variable correspond to the scores at the top of the nomogram). After summing these scores to obtain a total score, the predicted probability of an individual experiencing the outcome event can be determined by locating the total score on the prediction line at the bottom of the nomogram.

Using a random seed, the dataset was split into a training set and a validation set in a 7:3 ratio, with 231 cases in the training set and 100 cases in the validation set. Bootstrap resampling (1,000 iterations) was performed for internal validation with optimism correction. The optimism-corrected C-index was 0.823, indicating good discriminative ability; the calibration slope was 0.998, the calibration intercept was −0.005, and the Brier score was 0.169. These results indicate that the model exhibits good calibration and stability with minimal overfitting. Analysis of the ROC curve for the training set showed that the model's optimal cutoff value was 0.493, with a sensitivity of 85.4%, a specificity of 70.3%, and an AUC of 0.835 (95% CI: 0.783–0.886), indicating that the model has good predictive performance (see Figure 2(A)). Meanwhile, the model's performance remained stable in the validation set: AUC was 0.822 (95% CI: 0.735–0.909), accuracy was 76.2% (95% CI: 0.677–0.835), sensitivity was 73.8% (95% CI: 0.609–0.842), and specificity was 79.5% (95% CI: 0.635–0.907). The differences in all metrics between the training and validation sets were less than 3%, indicating that the model has good generalization ability. See Figure 2(B). The Hosmer-Lemeshow test yielded *χ*^2^ = 14.989 and *P* = 0.059; since *P* > 0.05, the difference between the model's predicted values and the observed values is not statistically significant, suggesting that the model has good predictive performance. Calibration plots are shown in Figures 3.

**Figure 1:**
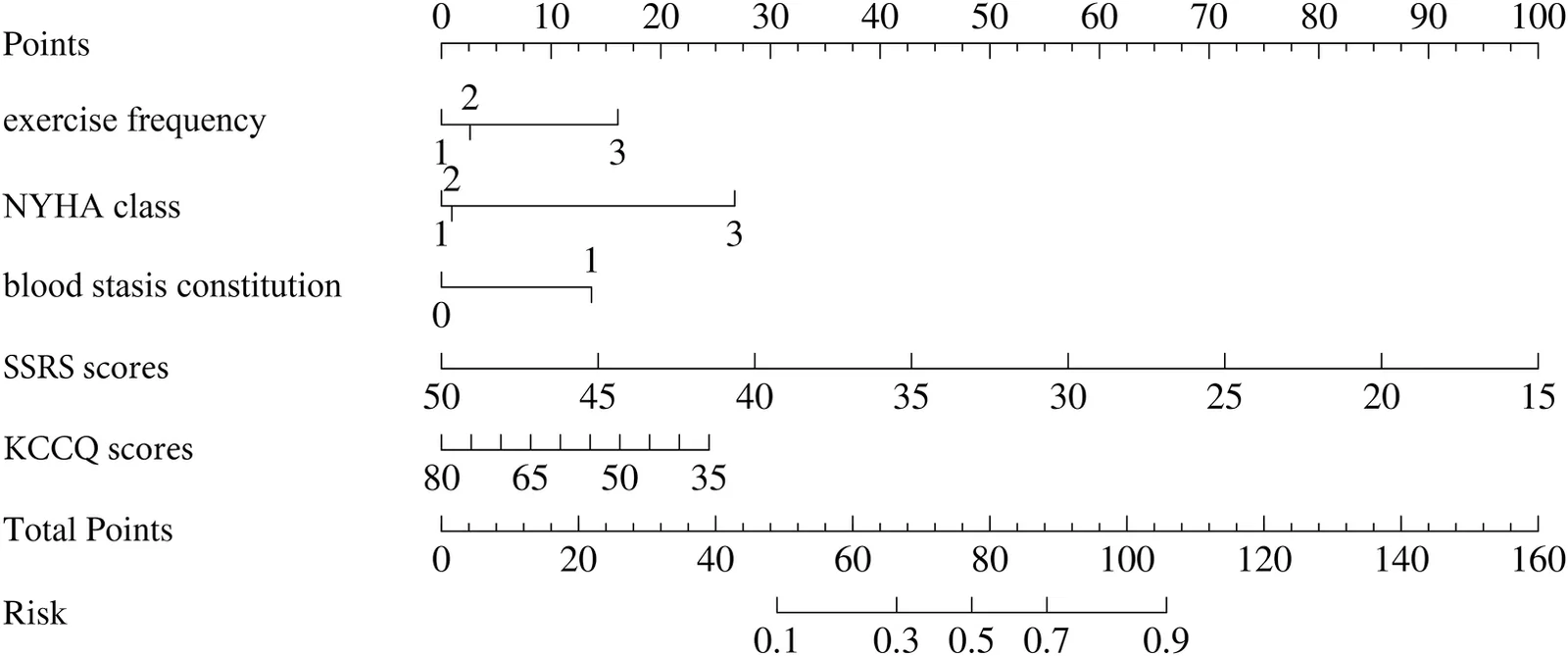
Nomogram for predicting demoralization syndrome risk in CHF patients. In this nomogram, each variable is assigned a point score on the top scale (Points). The total points are summed and projected onto the bottom scale (Total Points), corresponding to a predicted risk (Risk) ranging from 0.1 to 0.9. Variables include: exercise frequency (coded as 1 = low, 2 = moderate, 3 = high), NYHA functional class (coded as I and II = 1, III = 2, IV = 3), blood stasis constitution (coded as 0 = no, 1 = yes), SSRS scores (social support), and KCCQ scores (quality of life). To use the nomogram, locate the patient’s value on each variable axis, draw a vertical line upward to the Points axis to obtain the individual point score, sum all points, and then read the corresponding predicted risk at the bottom Risk scale.

**Figure 2:**
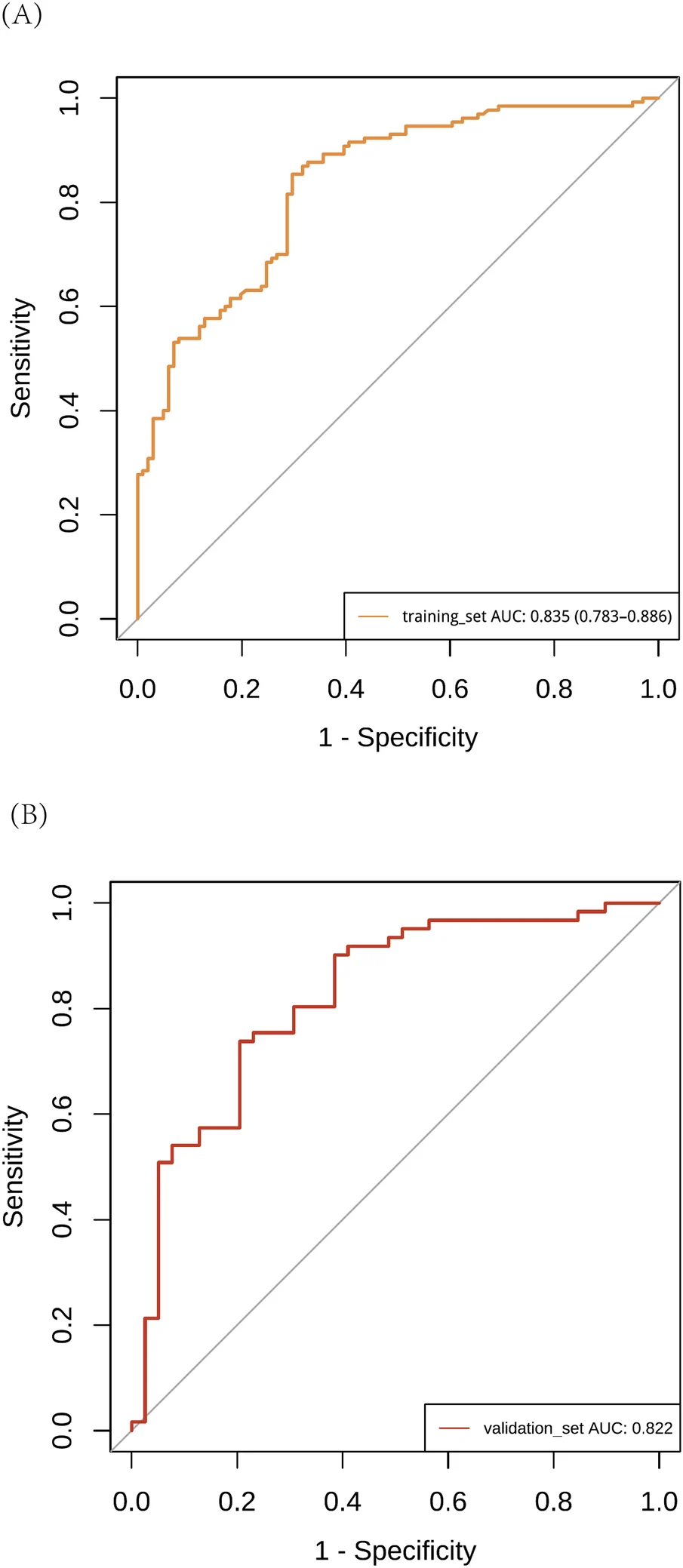
**(A)** ROC curve of the training set. **(B)** ROC curve of validation set.

**Figure 3:**
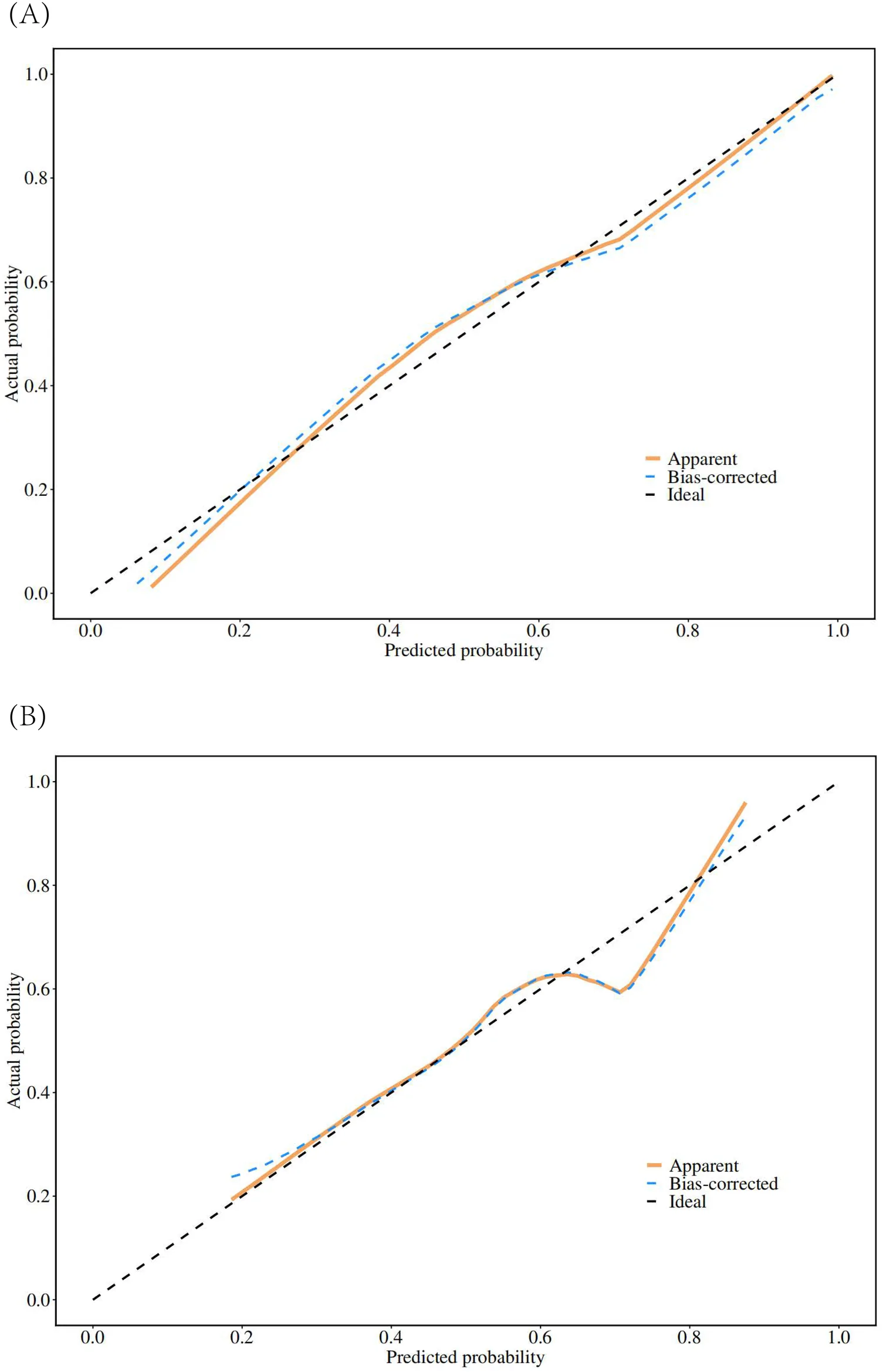
**(A)** calibration plot for the training dataset. **(B)** Calibration plot for the validation dataset. The *x*-axis represents the predicted probability, and the *y*-axis represents the observed probability. The diagonal dashed line indicates perfect calibration (ideal prediction). The solid orange line shows the actual performance of the nomogram, with error bars indicating 95% confidence intervals. Closeness of the orange line to the diagonal suggests good calibration. The loess-smoothed line and the histogram at the bottom show the distribution of predicted risks.

According to the DCA (Decision Curve Analysis) curve, the model yields a net benefit across most probability thresholds, indicating that it has good clinical applicability. See Figure 4.

**Figure 4:**
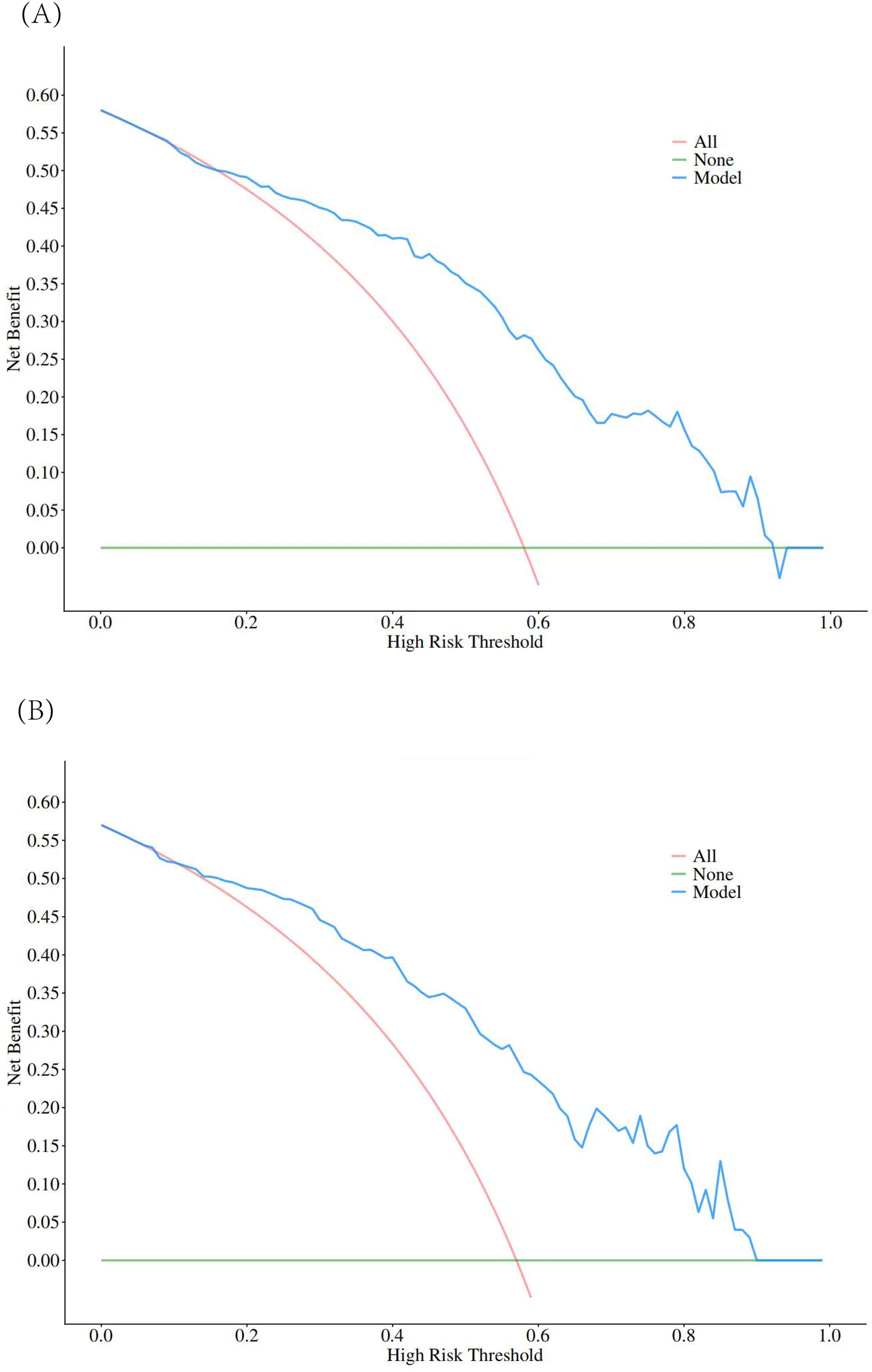
**(A)** DCA curves for the training dataset. **(B)** DCA curves for the validation dataset. The *y*-axis shows the net benefit, and the *x*-axis shows the threshold probability. The “Nomogram” curve (red) represents the net benefit of using the prediction model across various risk thresholds. The “All” curve assumes all patients are high risk and treated; the “None” curve assumes no patients are treated. A model is clinically useful if its net benefit exceeds the “All” or “None” strategies across a range of clinically reasonable thresholds.

## 4 Discussion

### 4.1 Key findings and model performance

This study enrolled 331 patients with chronic heart failure through a cross-sectional survey, of whom 191 (57.7%) presented with demoralization syndrome. Based on univariate and multivariate logistic regression analyses, five independent predictors were ultimately identified: exercise frequency,NYHA class, Social Support Rating Scale (SSRS) score, Kansas City Cardiomyopathy Questionnaire (KCCQ) score, and blood stasis constitution. The risk prediction model constructed using these factors had an AUC of 0.835 (95% CI: 0.783–0.886) in the training set, with a sensitivity of 85.4% and a specificity of 70.3%; in the validation set, the AUC was 0.822 (95% CI: 0.735–0.909), with a sensitivity of 73.8% and a specificity of 79.5%, suggesting that the model possesses good discriminatory power. The Hosmer-Lemeshow test (*χ*^2^ = 14.989, *P* = 0.059) and the calibration curve demonstrated a high degree of consistency between the model's predicted probabilities and the observed values, while receiver operating characteristic (ROC) curve analysis also indicated that the model offers net benefit across a wide range of threshold probabilities. These findings suggest that the model exhibits favorable predictive performance in internal validation and may constitute a promising tool for the early identification of patients with chronic heart failure at high risk for demoralization syndrome. Nevertheless, external validation is warranted prior to its implementation in clinical practice.

### 4.2 The prevalence and clinical significance

Currently, research into demoralization syndrome has primarily focused on cancer patients, with relatively limited studies in the field of chronic heart failure. In the existing literature, only a few studies have reported scores on the demoralization syndrome scale in heart failure patients, but none have explicitly reported the specific prevalence of demoralization syndrome. Gao et al. (19). surveyed 245 elderly patients with chronic heart failure; the results showed a DS-II score of (16.55 ± 4.76) points, with 21 patients (8.57%) scoring ≤9 points and 68 patients (27.76%) scoring ≥20 points; Gao et al. (20). used the revised Chinese version of the Demoralization Scale II to survey 282 patients with chronic heart failure, yielding a mean score of (30.30 ± 10.37) points. This study found that 57.7% of patients exhibited moderate to severe demoralization syndrome, suggesting that demoralization syndrome is quite common among patients with chronic heart failure and warrants clinical attention. The clinical manifestations of demoralization syndrome (such as fatigue, weakness, low mood and loss of interest) overlap significantly with the psychosomatic symptoms of heart failure itself, and are easily confused with negative emotions such as anxiety and depression, leading to clinical underdiagnosis or misdiagnosis. Demoralization syndrome is not only a direct manifestation of the patient's psychological distress, but also an independent predictors affecting treatment adherence, quality of life and prognosis. Without timely identification and intervention, patients may fall into a vicious cycle wherein ‘worsening symptoms exacerbate the severity of demoralization syndrome, which in turn undermines confidence in treatment, ultimately leading to a poorer prognosis’. Therefore, clinical healthcare professionals should consciously screen patients’ psychological state as part of routine heart failure management. Early identification and timely intervention not only help to improve patients’ mental health but are also a crucial component in enhancing the quality of comprehensive heart failure management and achieving ‘integrated mind-body care’.

### 4.3 The mechanisms underlying each predictor

Compared with patients who exercised daily, those who exercised rarely had a significantly higher risk of developing demoralization syndrome (OR = 3.47, 95% CI: 1.43∼8.39); however, there was no statistically significant difference between occasional and daily exercise, suggesting that there may be a dose-response relationship with exercise. Exercise can improve mood by modulating the levels of neurotransmitters such as dopamine, serotonin and endocannabinoids. A systematic review by Adilson et al. (21). (incorporating 15 studies) indicated that exercise not only increases dopamine release and receptor availability, but also reduces the risk of anxiety and depression through mechanisms such as improved cognitive function, elevated levels of brain-derived neurotrophic factor and enhanced brain plasticity. Clinical practice guidelines in the United States, the United Kingdom and Australia all explicitly recommend exercise as an integral component of depression treatment (22–24). For patients with chronic heart failure, the condition is essentially a systemic inflammatory state. Elevated levels of inflammatory mediators not only exacerbate myocardial damage and ventricular remodelling but can also affect the central nervous system via neuro-endocrine-immune networks, inducing or exacerbating negative emotions. Exercise can break this vicious cycle by suppressing the expression of inflammatory factors and enhancing anti-inflammatory responses. It is therefore recommended that individualised exercise programmes be developed for patients with chronic heart failure, preferably involving moderate-intensity aerobic exercise, to gradually establish a regular exercise routine.

This study identified the New York Heart Association (NYHA) functional class and the Kansas City Cardiomyopathy Questionnaire (KCCQ) score as independent risk and protective factors, respectively, for demoralization syndrome in patients with heart failure.

NYHA functional class is a core clinical indicator of heart failure severity. Higher NYHA class is associated with a progressively greater burden of cardinal symptoms, including dyspnea, fatigue, edema, and anorexia. These symptoms are not merely somatic phenomena; rather, they exert a profound influence on patients’ illness perception and psychological adaptation through complex psychophysiological interplay. Specifically, the escalating symptom burden may indirectly precipitate demoralization by reinforcing patients’ sense of fatalism—the entrenched belief that personal effort is futile in altering disease trajectory. Empirical evidence supports this mediating pathway: symptom burden exerts a significant negative effect on self-management behaviors via fatalism, with more severe symptoms fostering a fixed conviction that “no amount of effort can change the current situation,” thereby predisposing patients to demoralization syndrome (25). On the basis of this mechanistic framework, active symptom management and effective reduction of physical burden constitute the foundational intervention for mitigating demoralization in this population.

In addition, social support, as measured by the Social Support Rating Scale (SSRS), emerged as a protective factor against demoralization, buffering the development of negative affective states. Social support alleviates feelings of helplessness and isolation by providing both emotional reassurance and tangible assistance. Patients with heart failure, however, are particularly vulnerable to disconnection from their established social networks due to progressive symptom exacerbation, diminished physical capacity, and frequent hospital readmissions—all of which may potentiate demoralization. Notably, peer support combined with psychological stress intervention has been shown to significantly reduce psychological distress, enhance psychological resilience, and improve quality of life in patients with advanced heart failure (26).

This study identified blood stasis constitution as an independent predictor of demoralization syndrome in patients with chronic heart failure (OR = 2.88, 95% CI: 1.23∼6.72). Blood stasis constitution is relatively prevalent among older adults and represents a high-risk constitutional factor for various diseases. Individuals with this constitution typically present with dull or hyperpigmented skin, easy bruising and pain, dark or purplish lips, a purplish tongue or one with petechiae and ecchymosis, sublingual varicose veins, and a thready, unsmooth pulse (27). They may also exhibit irritability and forgetfulness. This constitution often arises from congenital predisposition, acquired injuries, or qi stagnation due to emotional depression, as well as from prolonged illness affecting the meridians. In terms of etiology and pathogenesis, heart qi deficiency is regarded as the root, with blood stasis and collateral obstruction as its manifestations. Since the heart governs the blood vessels, insufficiency of heart qi leads to sluggish blood flow, resulting in stasis—a pathological process of “deficiency leading to stasis.” Once established, blood stasis further impedes qi and blood circulation, exacerbates heart qi deficiency, and perpetuates a vicious cycle. Some studies suggest that emotional distress, liver qi stagnation, and constrained qi movement are key contributors to blood stasis (28). When qi and blood fail to nourish the heart, the heart loses its spiritual support, thereby worsening emotional disturbances. The core feature of demoralization syndrome is a subjective sense of helplessness. From the perspective of Chinese medicine, this helplessness is closely related to heart qi deficiency and collateral stasis obstruction. Because the heart governs both the blood vessels and mental activities, blood stasis obstructing the collaterals and depriving the heart of spiritual nourishment manifests as listlessness, low mood, and diminished coping ability. Thus, blood stasis constitution serves as a central link between chronic heart failure and demoralization syndrome. This suggests that, in clinical practice, therapies for activating blood and resolving stasis may be used as adjuncts to conventional heart failure treatment to achieve “simultaneous regulation of body and mind.” However, the relatively wide 95% confidence interval (1.23∼6.72) indicates limited precision of this estimate, and these findings warrant validation in larger, more diverse populations.

### 4.4 Clinical implications

Based on the above findings, this study proposes the following clinical recommendations:

1. The nomogram may serve as a triage tool for busy clinical settings. For example, a patient with infrequent exercise, NYHA class IV, low SSRS and KCCQ scores, and a blood stasis constitution would have a high predicted probability (>80%) of demoralization, flagging the need for immediate, in-depth psychological assessment using the DS-II. Conversely, a low predicted probability could help reassure clinicians and avoid unnecessary referrals. However, this application is preliminary and requires prospective validation.
2. Implement individualised comprehensive intervention strategies. For patients with insufficient physical activity, prescribe moderate-intensity aerobic exercise (e.g., 30 min of brisk walking daily) and gradually establish an exercise routine. For patients with insufficient social support, encourage family members to participate in care and consider introducing peer support programmes. For patients with blood stasis constitution, in addition to standard heart failure treatment, the combination of traditional Chinese patent medicines that promote blood circulation and remove stasis (such as Xuefu Zhuyu Capsules or Danlou Tablets) may be considered; however, these must be used under the guidance of a TCM practitioner, and attention must be paid to drug interactions. Application scenarios of the predictive model.
3. The nomogram developed in this study showed promising predictive performance in internal validation and may serve as an auxiliary reference for risk awareness in future clinical practice, pending successful external validation. The nomogram incorporates five readily available clinical variables: physical activity level, NYHA functional class, blood stasis constitution (yes/no), and two standardized scale scores (SSRS and KCCQ). However, given the observational nature of this study and the lack of external validation, this nomogram is not yet ready for independent clinical decision-making and should be used with caution until further validated in multicenter prospective cohorts.

## 5 Conclusion

Based on cross-sectional survey data, this study developed and internally validated the first risk prediction model for demoralization syndrome in patients with chronic heart failure. Exercise frequency, NYHA functional class, social support, symptom burden, and blood stasis constitution were identified as independent predictors of this syndrome. The model demonstrated good discriminatory power (AUC = 0.835), calibration, and clinical net benefit. Pending external validation, it may serve as a preliminary auxiliary reference for risk awareness in clinical practice. Future work should involve multi-centre prospective studies to further validate the model's external validity and to explore the effectiveness of intervention strategies based on this model in improving patient outcomes.

## 6 Limitations

(1) The cross-sectional design limits the inference of causal relationships, as all variables were measured simultaneously. (2) The sample was drawn from convenience sampling at two hospitals in Anhui Province, limiting its generalisability. (3) Internal validation was conducted using only random splitting (7:3) and the Bootstrap method; external validation on an independent external population was lacking. (4) For individuals with mixed constitutions, the lack of a uniform standard for designating the constitution with the highest conversion score as the primary type may overlook the interactive information between mixed constitutions, which may oversimplify the complex interplay among different constitutional imbalances, potentially obscuring the combined effect of, for instance, Qi stagnation and Blood stasis on demoralization. (5) Important variables such as history of mental illness, family functioning, medication adherence and personality traits were not included, suggesting the potential presence of unmeasured confounding factors. (6) Although scales with good reliability and validity were used, self-report measures may still be subject to social desirability bias or recall bias.

## Data Availability

The raw data supporting the conclusions of this article will be made available by the authors, without undue reservation.
